# Evaluation of the Bioaerosol Inactivation Ability of Chitosan-Coated Antimicrobial Filters

**DOI:** 10.3390/ijerph18137183

**Published:** 2021-07-05

**Authors:** Ying-Fang Hsu, Chi-Yu Chuang, Shinhao Yang

**Affiliations:** 1 Environmental Sustainability Lab, Center for General Education, CTBC Business School, No. 600, Section 3, Taijiang Boulevard, Annan District, Tainan 709, Taiwan; hsuyf@ctbc.edu.tw; 2 Department of Occupational Safety and Health, Chang Jung Christian University, No. 1, Changda Road., Gueiren District, Tainan City 711, Taiwan

**Keywords:** chitosan-coated filters, survival, bioaerosols, antimicrobial agent

## Abstract

This work considers the enhancement of indoor bioaerosol removal efficiency by liquid coating of the antimicrobial agent chitosan onto polypropylene fibrous filters (CCFs). *Escherichia coli* (*E. coli*) and *Bacillus subtilis* (*B. subtilis*) were chosen as the tested bioaerosols. The results revealed that 2.5% (*w*/*w*) of CCFs have significantly higher bioaerosol survival capability (23% and 34% of *E. coli* and *B. subtilis*, respectively), compared to an untreated filter (65% and 64% for *E. coli* and *B. subtilis*, respectively). Increasing face velocity and relative humidity during operating CCFs could reduce the bioaerosol removal capability. The regression analysis of the experimental findings demonstrated that the higher coating concentration of chitosan had the most positive influence on bioaerosol removal, while the face velocity and relative humidity had a negative influence, but a milder effect was observed (R^2^ = 0.83 and 0.81 for *E. coli* and *B. subtilis* bioaerosols, respectively). A CCF-loaded air-cleaning device was tested in a real indoor environment and resulted in 80.1% bioaerosol removal within 3 h of operating, which suggests that the chitosan-coated filter has the potential for further application in improving indoor air quality in the future.

## 1. Introduction

The investigation displayed that modern people spend an average of 87.2% of their time indoors. Thus, indoor air quality is a significant environmental and health issue [1]. Among the indoor contamination sources, airborne microorganisms, the major composition of bioaerosols, can cause various respiratory problems, allergic syndromes, and infectious diseases, as well as have a serious impact on indoor air quality [2,3]. Therefore, many air-cleaning techniques, including ozone releasing, photocatalytic oxidation, ultraviolet germicidal irradiation, plasma neutralization, and disinfectant spraying, have been developed for inactivating indoor airborne microbial contamination.

Li and Wang [4] investigated the inactivating ability of the ozone on bioaerosols. Their results demonstrated that when the ozone concentration was increased to 10 ppm, the germicidal efficiency toward the bacteria and the fungi bioaerosols began to become obvious. Lin and Li [5] examined the effectiveness of titanium dioxide photocatalyst filters under a wavelength of 365 nm ultraviolet light in controlling bioaerosols. They found that the effect of photocatalytic oxidation on bioaerosol removal was weak. Lin and Li [6] employed ultraviolet germicidal irradiation to remove indoor bacteria and fungi bioaerosols. Their findings indicated that ultraviolet germicidal irradiation has the best removal efficiency for *Escherichia coli* (*E*. *coli*) bioaerosols and the poorest ability to control spores of *Penicillium citrinum.* When the ultraviolet germicidal irradiation was applied to the indoor environment for 10 min, the concentration of environmental bioaerosols decreased greatly. However, the ultraviolet germicidal irradiation may also generate a secondary pollutant (ozone and nitrogen oxides) and subsequently cause the air quality to deteriorate. Yang et al. [7] investigated the ability of a carbon nanotube (CNT) corona discharge plasma system to inactivate bioaerosols. The inactivation efficiencies of *E. coli*, *B. subtilis,* and λ virus bioaerosols using CNT corona discharge plasma were as high as 93%, 88%, and 81%, respectively. Chuang et al. [8] indicated that spraying electrolyzed water containing as high as 200 ppm free available chlorine was revealed to be significantly effective in the inactivation of bacterial airborne contamination.

Although all of the above-mentioned available air-cleaning technologies are effective in bioaerosol inactivation, environmentally friendly (such as the minimum disinfectant residual) standards and personal safety requirements cannot be fulfilled. On the other hand, filtration, as the most traditional air-cleaning technology, has the strengths of being non-residual and safe for humans. In addition, some studies are pursuing the improved efficiency of non-woven air filters in capturing bioaerosols. Shi et al. [9] indicated that the polypropylene electret filter is highly efficient in removing bacterial aerosol from air. In Yang’s study [10], an air filter was pretreated with electric charge and surfactant to increase its capacity to capture *E. coli* and yeast bioaerosols. In addition, Yang’s study indicated that indoor environment parameters, such as face velocity and relative humidity, influence the removing efficiency of bioaerosols due to shorting retention time and surfactant activity, respectively. Recently, air-filtering media treated with natural antimicrobial agents have been in high demand as they can achieve desirable collection efficiency and self-deactivate the viability of bioaerosols, eventually resulting in a reduced biocontamination risk in the indoor environment.

Hereby, this work developed an easily prepared, low-cost, and antimicrobial filter for inactivating indoor bacterial bioaerosols. Chitosan is a natural biopolymer composed of two chemical elements, randomly distributed β-(1→4)-linked D-glucosamine and N-acetyl-D-glucosamine. Chitosan has been used in a variety of industries, including medical, food, textiles, cosmetics, agriculture, and chemical engineering. Many studies have indicated that chitosan coated into textile fibers or added into liquid is an acceptable antimicrobial agent, which has a significant ability to inactivate microorganisms [11,12,13,14,15,16,17,18]. Ozden and Basal (2017) applied polyamide 6/chitosan nanofiber-coated on HEPA filter for controlling bioaerosols [19]. Chen et al. (2019) used zero-valent nano-silver/Ti0_2_-chitosan composite filters to remove hospital indoor bioaerosols [20]. Mishra et al. (2021) applied lemongrass essential oil loaded on chitosan nanocellulose for inactivating bioaerosols [21]. These above studies used chitosan’s antibacterial ability to inactivate bioaerosols. However, the applications of chitosan in these studies were coating chitosan on a high-efficiency HEPA filter or combining chitosan with multiple antibacterial ingredients on a filter.

Therefore, this work aimed to develop an antimicrobial filter by using the low-efficiency filter coated with the antimicrobial agent chitosan, characterizing its overall removal performance in removing biological and non-biological aerosols. The addition of the biological inactivating effects of a chitosan coating were clarified by comparing the overall removal performance among biological and non-biological aerosols (tested and expressed as penetration rate). First, this work utilized standard, non-biological latex aerosol to test the newly developed chitosan-coated filters (CCFs) to evaluate their physical aerosol penetration rate. Second, based on the physical aerosol penetration rate of non-biological aerosols, *E. coli* and *Bacillus. subtilis* (*B. subtilis*) were then selected as the analyzed bioaerosols to elucidate their biological inactivating ability, examined by combining physical penetration with survival conditions through CCFs. Two essential environmental factors that majorly affect filtering performance, relative humidity and face velocity, were included in the study.

## 2. Materials and Methods

### 2.1. Filter Media

The characteristics of the non-antibacterial original filter and CCFs are both presented in Table 1. A commercial single-layer polypropylene (PP) fibrous air filter was purchased as the original untreated media in the filter (KNH Enterprise Co., Ltd., Tainan, Taiwan). The antibacterial CCFs were prepared by using original polypropylene fibrous filters coated with chitosan at various concentrations (1.0%, 1.5%, and 2.5%, *w*/*w*). The chitosan-coating solutions were made by dissolving chitosan (Merck & Co., Inc., Kenilworth, NJ, USA) in pure water. The coating procedure of the CCFs is shaking (50 rpm) the original filter media with chitosan solution for 1 min (under 30 °C) to sufficiently coat the chitosan on the filter fiber. The chitosan solution was mixed completely and the time the filter soaked in the solution was long enough for chitosan coating on the fiber surface. After the coating procedure, the CCFs were dried in a condition of 105 °C for 12 h. The uniformity of CCFs was very good in this study. According to the observation of scanning electron microscope (SEM), the coated fiber diameters were nearly the same, and the chitosan was coated on fiber surface uniformly.

### 2.2. Tested Bioaerosols

According to the previous studies, *E. coli* and *B. subtilis* were chosen as the analyzed bioaerosols for indoor bioaerosol inactivating tests [22,23]. *E. coli*, a Gram-negative bacterial strain, was selected to represent human activity-related indoor environmental bacterial species. On the other hand, *B. subtilis,* a Gram-positive endospore-forming bacterial strain, commonly found in soil and rotten plants, was selected to represent bio-contaminants from the atmospheric environment in this study. Following previous laboratory-based bioaerosol studies [6,7,8,24], two different types of bioaerosols were analyzed individually to understand their specific survival capability against antimicrobial agents using the culture method. Accordingly, the vegetative cells of *E. coli* (Bioresource Collection and Research Center in Taiwan, BCRC 10675) and *B. subtilis* (Culture Collection & Research Center in Taiwan, CCRC 12145) were purchased and activated for the preparation of bioaerosols based on our previous study [7]. Three *E. coli* and three *B. subtilis* colonies were transferred from the individual pure culture agar plate and cultivated into a conical flask containing 30 mL of tryptic soy broth (TSB, Bacto™). The transferred colonies in the TSB were cultivated under a shaking condition of 85 rpm, for 16–24 h at 37 °C, for enrichment. After the enrichment, the TSB solution was centrifuged under the condition of 2500 rpm for 5 min, and the resulting supernatant was removed. Then, the *E. coli* and *B. subtilis* sediments were re-suspended by adding 30 mL of phosphate buffered saline solution (PBS, pH 7.2). These processes were repeated twice to remove TSB medium. Finally, this PBS solution was used for bioaerosol generation. The initial concentration of both bacterial strains in the solution was determined as 10^5^ CFU/mL with serial dilutions. Then, the bacterial solution was loaded to the Collison three-jet nebulizer (BGI Inc., Waltham, MA, USA) and included in the experimental setup (described in Section 2.4). When supplied with 20 psig clean and compressed air, the nebulizer generates *E. coli* and *B. subtilis* bioaerosol streams in a range of 4 to 8 × 10^4^ CFU/m^3^.

### 2.3. Tested Non-Biological Aerosols

This work applied a monodisperse polystyrene latex (PSL, Duke, CA, USA) sphere particle (geometric diameter: 0.9 μm; GSD: 1.02; presents similar aerodynamic behavior with *E.coli* and *B. subtilis* bioaerosols) as the testing aerosol to understand the efficiency with which aerosols penetrate the CCF filters according to previous studies [25,26]. The PSL aerosols were generated using the Collison three-jet nebulizer (BGI Inc., Waltham, MA, USA), and measured by an Aerodynamic Particle Sizer (APS, model 3320, TSI Inc., Shoreview, MN, USA).

### 2.4. Aerosol Experimental Setup

**Figure 1:**
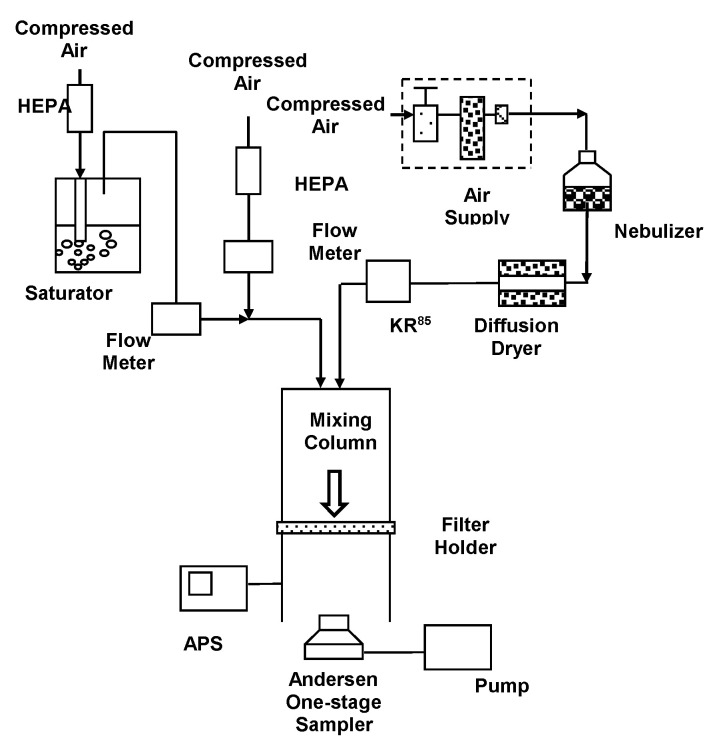
Schematic diagram of the experimental system.

Figure 1 schematically describes the aerosol experimental setup. This work utilized to test the physical removal efficiency and biological inactivating ability through the CCFs. The aerosol experimental setup includes the Collison three-jet nebulizer, the airflow mixing column, the testing filter holder (100 mm diameter, to support the CCFs filter and maintain its integrity during aerosols stream passing), the Anderson one-stage impaction sampler (Andersen Samplers, Inc., Atlanta, GA, USA), APS, and flowmeters.

The analyzed aerosols (namely, non-biological and biological) were aerosolized by the Collison three-jet nebulizer (BGI Inc., Waltham, MA, USA) and dried by passing them through a diffusion dryer as a stream. The dried aerosol stream was be neutralized and checked by using a Kr-85 radioactive source (model 3077, TSI Inc.) and an aerosol electrometer (model 3068, TSI Inc.). The analyzed aerosols were mixed with diluted clean air and flowed into the mixing column. In this work, three face velocities (10, 20, and 30 cm/s) through the tested filter were selected. The face velocity through the tested filter was controlled by the diluted clean air flow (face velocities = Q/A, Q equal to analyzed aerosols flow + diluted clean air flow; A equal to tested filter area). In this work, we selected three relative humidity (RH, 30%, 50%, and 70%) conditions to simulate the ideal indoor environment (RH range: 30–50%, according to U.S. EPA indoor mold control). A high humidity condition (RH 70%) was also included in this work to simulate the indoor condition in a subtropical zone. The RH of the analyzed aerosol flow in the mixing column was controlled by changing the ratio of the flow rate of humidified gas stream generated by the water vapor saturator and dry gas stream. The RH of analyzed aerosol flow were sampled using a hygrometer (Hygromer A2, Rotronic Inc., Hauppauge, NY, USA).

The physical aerosol penetration efficiency of the testing filter was evaluated by analyzing the PSL aerosol. The physical aerosol penetration efficiency (P_Std_, %) was defined as the ratio of C_with_/C_without_, where C_with_ represents the physical aerosol counting concentrations (numbers/m^3^) sampled from the downstream of the filter holder installed with a CCF filter using an APS. C_without_ represents the physical aerosol counting concentrations (numbers/m^3^) sampled from the downstream of the filter holder without a CCF filter measured by APS.

Considering the biological inactivating effects of chitosan coating, the filter was evaluated for its survival recovery of bioaerosols (R%, R_Ec_ indicates the survival of *E.coli* and S_Bs_ indicates the survival of *B. subtilis*), which was calculated in the ratio of S_with_/S_without_, where S_with_ represents the bioaerosol culturable concentrations (CFU/m^3^) sampled from the downstream of the filter holder installed with a CCF filters using an Andersen one-stage impaction sampler. S_without_ represents the culturable concentrations (CFU/m^3^) sampled from the downstream of the filter holder without a CCF filter and measured using an Andersen one-stage sampler. The bioaerosol collection flow rate of the sampler was set at 28.3 lpm based on the operation manual. Bacterial bioaerosols were collected on tryptic soy agar (TSA, Difco Laboratories, Detroit) and cultured at 37 °C for 24 h.

## 3. Results and Discussion

### 3.1. Non-Biological Aerosol Removal through CCFs

**Figure 2:**
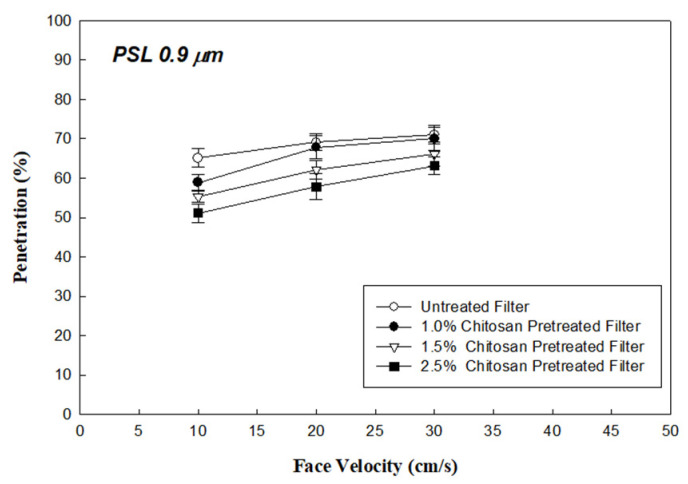
Penetration through the chitosan pretreated and untreated filters for 0.9-μm PSL aerosols.

Figure 2 shows the P_Std_ of 0.9 μm PSL aerosols through the uncoated single-layer PP material filter, and the 1.0%, 1.5%, and 2.5% CCFs, at face velocities of 10, 20, and 30 cm/s (RH 30%), respectively. The results showed the aerosol penetrations through the untreated filter, and the 1.0%, 1.5%, and 2.5% CCFs at a face velocity of 10 cm/s were approximately 63.1%, 58.9%, 55.3%, and 51.1%, respectively. The non-biological, standard PSL aerosol penetration through the three CCFs was lower than that of the original uncoated filter, up to 12% (from 63.1% to 51.1%, under 10 cm/s face velocity). Increasing the face velocity of the filters, on the contrary, minimized the difference between the aerosol penetration rates (from 68.2% to 59.1%, under 30 cm/s). These chitosan-coating addition effects also found that as the coated chitosan concentration increased, the aerosol penetration (P_Std_) decreased. Pretreating the filter with chitosan could increase the thickness of the filter and cause a higher interception effect toward aerosols. However, in this work, the observed result shows that pretreating the filter did not significantly alter the mechanical characteristics of the filter fiber (shown as Table 1). Therefore, this indicates that when the filter was coated with chitosan, the fiber surface properties were altered and resulted in an enhancement of the electrostatic aerosol-capturing forces, which resulted in a strong molecular polarity and positively-charged NH3^+^ group of chitosan. As a result, the physical collection efficiency (i.e., penetration prevention) of aerosols was increased. Additionally, this electrostatic enhancing effect was reduced when face velocity was increased. A similar finding was also observed in other studies, which applied polymers to air filters [27,28].

### 3.2. Survival Evaluation of Bacterial Bioaerosols through CCFs

**Figure 3:**
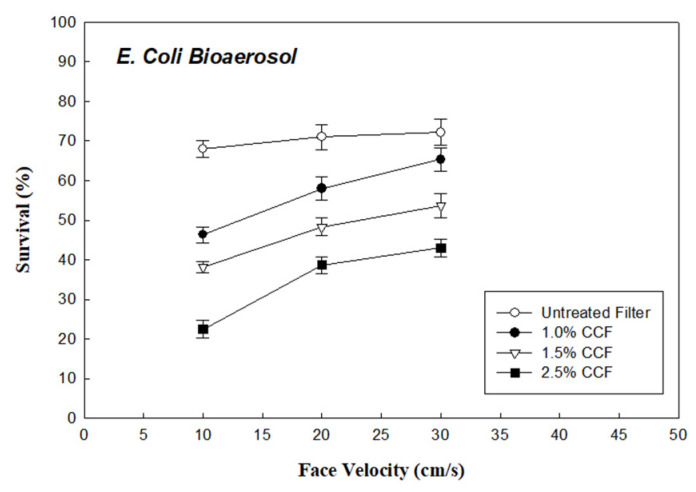
Survival of *E. coli* bioaerosols through the chitosan-coated and untreated filters.

Figure 3 shows the survival of *E. coli* bioaerosols (R_EC_) through the untreated filter, and the 1.0%, 1.5%, and 2.5% CCFs (under face velocity of 10 cm/s and RH of 30%). These data indicate that the R_EC_ of *E. coli* bioaerosols through the untreated filter, and the 1.0%, 1.5%, and 2.5% CCFs were approximately 65%, 46%, 38%, and 23%, respectively. Comparing these data with the results of P_Std_ of the PSL aerosol through these testing filters, it was found that the survival of the *E. coli* bioaerosol and the PSL aerosol through the untreated filter were similar (P_Std_ = 63.1% and R_Ec_ = 65%, under face velocity 10 m/s). However, the survival of the *E. coli* bioaerosol through the CCFs was much lower than that through the PSL aerosol, indicating that chitosan has an additional antibacterial effect on the *E. coli* bioaerosol.

The penetration (P_Std_) of the PSL aerosol through the CCFs revealed that the electrostatic force increased when the filter was coated with chitosan. In the filtration of the *E. coli* bioaerosol using CCFs, the enhanced electrostatic force and antimicrobial effects were added to the original capture force from the untreated filter. Accordingly, 28% of the variations in the survival (R_Ec_ = 23%, 2.5% CCFs under face velocity 10 m/s) of the *E. coli* bioaerosol and the PSL aerosol penetration (P_Std_ = 51.1%, 2.5% CCFs under face velocity 10 m/s) through the CCFs were attributed to the antibacterial effect of the filter pretreated with chitosan. In addition, the differences between R_Ec_ of the *E. coli* bioaerosol and the PSL aerosol penetration (P_Std_) through the 1.0%, 1.5%, and 2.5% CCFs were approximately 13%, 17%, and 28%, respectively. The experimental results reveal that a higher concentration of CCFs corresponds to a decrease in *E. coli* bioaerosol survival, resulting in a larger difference between *E. coli* (R_EC_ = 46% and 23% on 1.0% and 2.5% CCFs, respectively) and PSL (P_Std_ = 58.9% and 51.1% on 1.0% and 2.5% CCFs, respectively). These findings could be realized because the filter coated with a higher concentration of chitosan has a stronger antimicrobial effect against *E. coli* bioaerosols.

**Figure 4:**
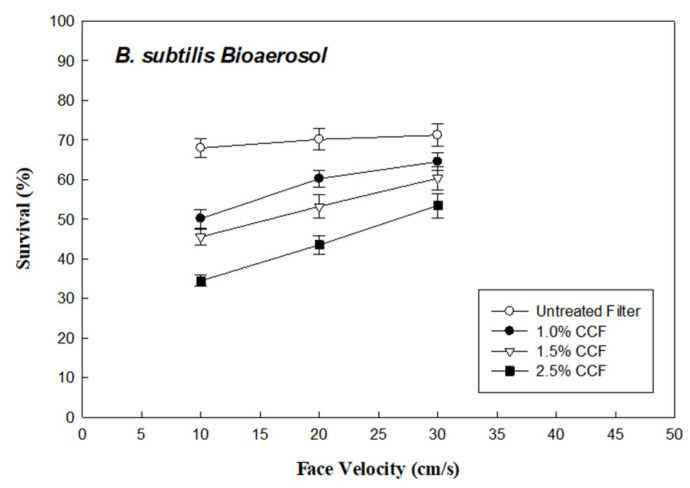
Survival of *B. subtilis* bioaerosols through the chitosan-coated and untreated filters.

Figure 4 shows the survival (R_Bs_) of *B. subtilis* bioaerosols through the testing filters under a face velocity of 10 cm/s and RH of 30%. The data reveal that the R_Bs_ of *B. subtilis* bioaerosols through the untreated filter, and the 1.0%, 1.5%, and 2.5% CCFs were approximately 64%, 50%, 46%, and 34%, respectively. The survival of the *B. subtilis* bioaerosol and the PSL aerosol through the untreated filter were similar (P_Std_ = 63.1% and R_Bs_ = 64%, under face velocity 10 m/s). However, the *B. subtilis* bioaerosols through the CCFs were much lower R_Bs_ than those of the PSL aerosol (R_Bs_ = 34% and P_Std_ = 51%, 2.5% CCFs under face velocity 10 m/s). This demonstrates that chitosan has a biological inactivating ability against the *B. subtilis* bioaerosol. The differences between the R_Bs_% of the *B. subtilis* bioaerosol and the PSL aerosol penetration (P_Std_) through the 1.0%, 1.5%, and 2.5% CCFs were approximately 9%, 10%, and 17%, respectively. It can be understood that variations in the survival of the *B. subtilis* bioaerosol (R_Bs_) and the PSL aerosol penetration (P_Std_) through the CCFs were attributed to the biological inactivating effect of the filter coated with chitosan. These findings, showing a similar trend to the *E. coli* bioaerosol tests, reveal that a filter coated with a higher concentration of chitosan results in a greater antimicrobial effect against *B. subtilis* bioaerosols.

Furthermore, these CCFs had a higher ability to inactive *E. coli* bioaerosols (R_Ec_ = 46%, 38%, and 23% with 1.0%, 1.5%, and 2.5% CCFs, respectively) than *B. subtilis* bioaerosols (R_Bs_ = 50%, 46%, and 34% with 1.0%, 1.5%, and 2.5% CCFs, respectively), mainly due to the fact that Gram-negative *E. coli* is a bacterial strain that is sensitive to antimicrobial agents, while Gram-positive *B. subtilis* is a resistant bacterial strain [22,23] with a mainly peptidoglycan-consisted cell wall structure. Therefore, compared with *B. subtilis* bioaerosols, *E. coli* bioaerosols are more antimicrobial agent sensitive and easily biologically inactivated and removed from indoor air by CCFs. This finding is consistent with the results of another bioaerosol control approach in a previous study of Lin and Li [6].

### 3.3. Effect of Face Velocity on Bioaerosol Survival through CCFs

Figure 3 shows the survival (R_Ec_) of the *E. coli* bioaerosols through the 1.0%, 1.5%, and 2.5% CCFs at face velocities of 10, 20, and 30 cm/s (RH 30%), respectively. The R_EC_ of the *E. coli* bioaerosols through the 2.5% CCF increased from 23% to 37% as the face velocity increased from 10 to 30 cm/s. As shown in Figure 4, the survival (R_Bs_) of *B. subtilis* bioaerosols through the 2.5% CCFs increased from 34% to 48% as the face velocity increased from 10 to 30 cm/s. The results display that the R_Ec_ of *E. coli* through these three CCFs increased with the face velocity, which indicates that the biological inactivating ability of the CCFs decreased with the increase in face velocity. This is probably due to the increase in face velocity (mechanical) reducing the residence time associated with bioaerosols attraction (the majorly related to electrostatic-capturing force, which is weaker than mechanical force) to the chitosan on the surface of the CCFs [26,27]. According to the experimental data, the antimicrobial effect on CCFs decreased as the face velocity increased, which could be attributed to the shorter residence time.

### 3.4. Effect of Relative Humidity on Survival through the CCFs

**Figure 5:**
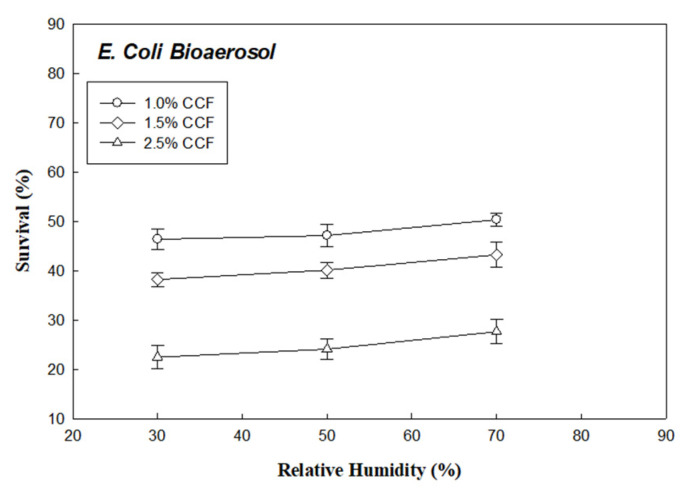
Survival of *E. coli* bioaerosols through the chitosan-coated and untreated filters in different relative humidity.

**Figure 6:**
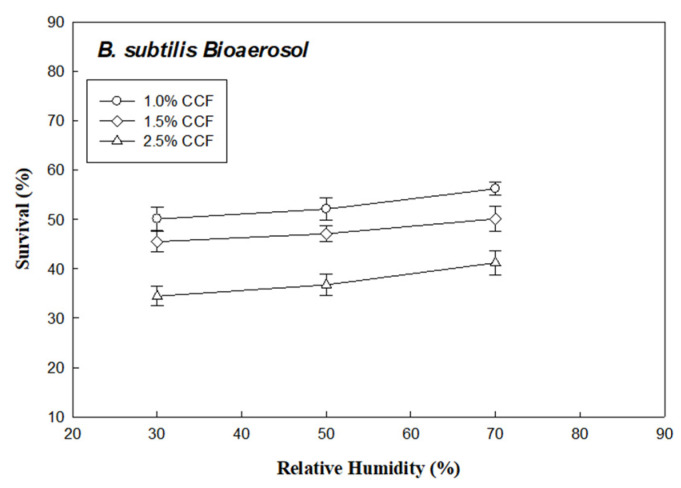
Survival of *B. subtilis* bioaerosols through the chitosan-coated and untreated filters in different relative humidity.

Figure 5 and Figure 6 show the plot of survival of *E. coli* (R_Ec_) and *B. subtilis* (R_Bs_) bioaerosols through the 1.0%, 1.5%, and 2.5% CCFs at RH values of 30%, 50%, and 70% (under face velocity of 10 cm/s), respectively. According to these results, the survival of the two bacterial bioaerosols (R_Ec_ and R_Bs_) through CCFs increased with the RH.

This is probably due to the fact that as the inactivating test was controlled under the condition of higher RH, the fiber surface of the CCFs accumulated a higher water content. Chitosan is hygroscopic and has a high capability to form hydrogen bonds. However, it might interfere with the electrostatic-capturing force (majorly generated by charged NH3^+^ group of chitosan) or alter the polymer’s physicochemical and biological properties [29,30]. This mechanism would decrease the biological inactivating ability of CCFs.

### 3.5. Application of CCFs in Field Indoor Environments

**Figure 7:**
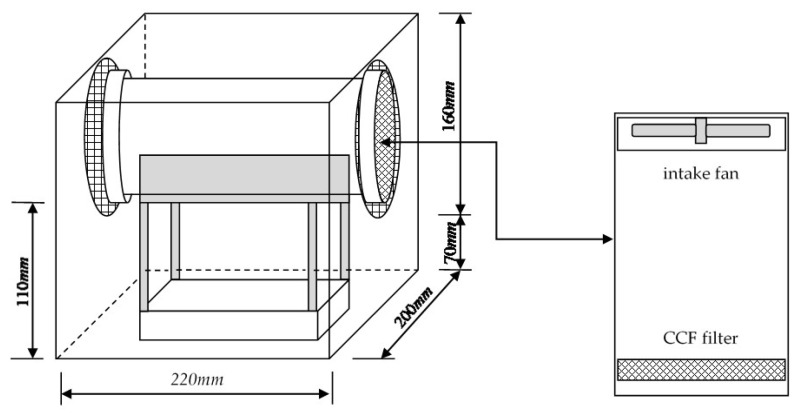
Schematic diagram of self-made air-cleaning device.

**Figure 8:**
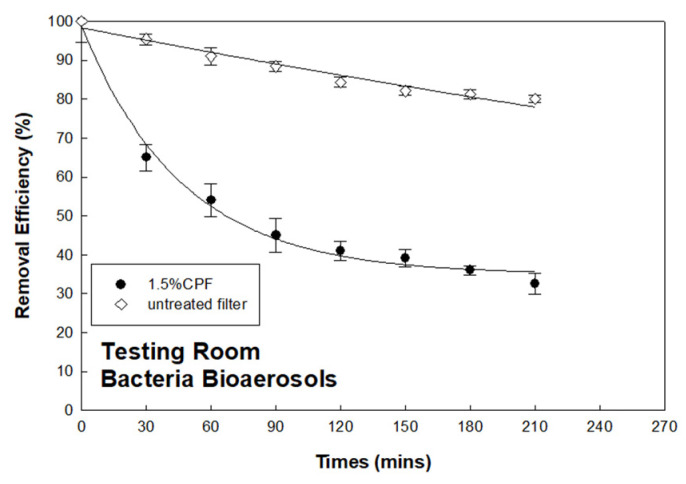
Survival of bacterial bioaerosols by using the 1.5% chitosan-coated and untreated filters in the testing room.

This work applied CCFs in a real indoor environment to understand their bioaerosol field removal capacity. A dental clinic was chosen as the testing room. The size of the testing room was 3 × 3 × 1.8 m^3^. A self-made air-cleaning device was fabricated with a 220 × 200 × 160 mm^3^ stainless-steel body-box and DC-powered fan (12V, diameter 40 mm, mounted in air intake side). A diameter 100 mm filter holder was installed on the opposite side of the body-box as an exhaust (shown as Figure 7). An untreated air filter or 1.5% CCF could be placed into the holder to clean the indoor air intake using a fan with 10 cm/s velocity. This self-made air-cleaning device was applied approximately 3 h after the end of a day-long dental operation. The bacterial bioaerosols were sampled in triplicate by an Andersen one-stage sampler at intervals of 30 min, followed by the collection and cultivation process described in Section 2.4. The background average bacteria bioaerosol concentration in this test room was monitored and calculated as 1513 ± 145 CFU/m^3^ before applying the air-cleaning device. Figure 8 shows that the bacteria bioaerosol removal efficiency when using the 1.5% CCFs and untreated filters in the testing room. During the testing process, the average RH was 55.5%, and average temperature was 25.6 °C. The experimental result indicates that the bacteria bioaerosol removal efficiencies when using the 1.5% CCFs and untreated filters were 32.6% and 80.1%, respectively. This preliminary, single-shot finding implies that the CCFs have potential for indoor air cleaning.

### 3.6. Evaluating the Effects of Operating Parameters on CCFs’ Bioaerosol Removal Characteristics

To characterize the bioaerosol removal performance of CCF, this work aimed to build a regression equation to integrate various parameters’ effects on the survival of bacterial bioaerosols through CCFs. The multiple regression analysis was used to understand the effects of different parameters on bioaerosol inactivating. This model was applied in our pervious study [31]. According to the results, the survival behaviors of *E. coli* and *B. subtilis* bioaerosols were obviously different due to their specific biological characteristics (Gram-positive and -negative).

Hence, two regression equations were calculated for these two bioaerosols, respectively. The regression equation considered the chitosan concentration (1.0%, 1.5%, and 2.5%), face velocity through the CCFs (10, 20, and 30 cm/s) and RH (30%, 50%, and 70%). The regression equation was constructed as follows.

S = aW^b^U^c^H^d^ (1)

where a, b, c, and d are the regression constants; S is the total survival rate through the CCFs; W is the chitosan concentration (%); U is the face velocity (m/s); and H is the RH (%). According to the regression analysis, the regression equations for each *E. coli* and *B. subtilis* bioaerosols are as follows:

S = 0.65W^0.25^U^−0.14^H^−0.08^ (2)

S = 0.38W^0.20^U^−0.11^H^−0.05^ (3)

Based on the regression analysis, the correlation coefficients R^2^ were approximately 0.83 and 0.81 for the regression equations for *E. coli* and *B. subtilis* bioaerosols, respectively. By comparing the coefficients in each equation, the effects of the different parameters on bioaerosol survival were demonstrated. The regression analysis results show that in each regression equation, the constant b is largest and indicates that the ‘‘chitosan-coating concentration’’ has the greatest influence on the inactivating ability of the CCFs among all influencing factors. According to these regression data, “face velocity” has the second highest impact on the survival of CCFs, and the effect of the RH on survival is the lowest, based on our experimental data. In this work, we chose *E. coli* and *B. subtilis* as the Gram-negative and Gram-positive analyzed bioaerosols, which are common and reliable options in bioaerosols studies. However, there are still many microorganisms in a broader sense of biological characteristics (such as yeast, fungi, and viruses). The degree of influence of these three factors only existed under the controlled condition of the experiment and was limited by the chosen bioaerosols.

## 4. Conclusions

Bioaerosols cause various adverse health effects and have a significant impact on indoor air quality. The air-cleaning technology must be effective, cost beneficial, environmentally friendly, and safe for humans. In this work, we attempted to enhance bioaerosol removal efficiency by means of simply coating a commercial air filter with chitosan. Within the laboratory experiments of non-biological PSL aerosol and bioaerosols (including *E. coli* and *B. subtilis*), the CCFs were proven to have additional removal capability due to their antimicrobial properties. Moreover, the installed CCF self-made air-cleaning device showed potential in removing bacterial bioaerosols in the field environment.

Among the operating parameters influencing CCF removal performance, we used regression analysis to show that the chitosan-coating concentration has the greatest influence. The face velocity and RH, on the other hand, have mild effects on bioaerosol removal. The approaches of simply preparing a chitosan-coated filter and performance characterization we used in this work will be further improved and utilized to test various antimicrobial agents and bioaerosols.

## Figures and Tables

**Table 1:** Fiber characteristics of original filters and CCFs.

Type	Material	Measured Weight of Filter (g/m^2^)	Measured Mean Fiber Diameter ^a^ (μm)	Measured Mean Filter Thickness ^b^ (cm)	Pa@10 cm/s
Untreated	Polypropylene	60.5 ± 0.1	10.1 ± 0.3	0.10 ± 0.01	5.1 ± 0.1
1.0% CCF	Polypropylene/ Chitosan	66.3 ± 0.2	10.2 ± 0.5	0.11 ± 0.00	5.1 ± 0.2
1.5% CCF	Polypropylene/ Chitosan	70.2 ± 0.3	10.3 ± 0.3	0.10 ± 0.01	5.1 ± 0.1
2.5% CCF	Polypropylene/ Chitosan	75.3 ± 0.2	10.2 ± 0.1	0.10 ± 0.01	5.1 ± 0.1

^a^ Diameter was measured by SEM experiments. ^b^ Filter thickness was measured by digital vernier; the mean fiber thickness was averaged by triple tests.

## Data Availability

The data supporting the results in the current study are available from the corresponding author on reasonable request.
